# Fifteen Years Later: Hard and Soft Selection Sweeps Confirm a Large Population Number for HIV In Vivo

**DOI:** 10.1371/journal.pgen.1004179

**Published:** 2014-02-20

**Authors:** Igor M. Rouzine, John M. Coffin, Leor S. Weinberger

**Affiliations:** 1 The Gladstone Institutes, Gladstone Institute of Virology and Immunology, University of California San Francisco, San Francisco, California, United States of America; 2 Tufts University, Sackler School of Biomedical Sciences, Boston, Massachusetts, United States of America; 3 HIV Drug Resistance Program, Center for Cancer Research, National Cancer Institute, Frederick, Maryland, United States of America; Imperial College London, United Kingdom

**Figure 1:**
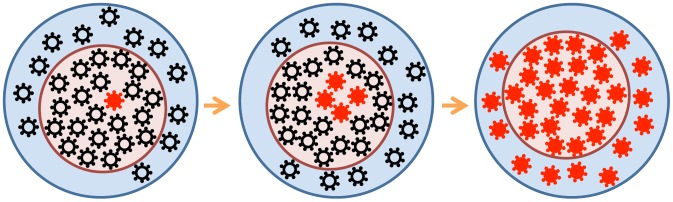
Beneficial viral mutants (red) arise in the “effective” virus subpopulation (*N*_eff_, pink circle) and spread gradually to the entire “census” population (blue circle). For a number of reasons (see the text), the effective population may be much smaller than the census population.

Even among RNA viruses, which generally exhibit high evolutionary plasticity due to low fidelity of their RNA polymerases, HIV-1 is second only to HCV for its ability to generate within-host genetic diversity [1]. HIV's rapid generation time leads to this high genetic diversity. The unfortunate consequences of HIV's rapid evolution are resistance to antiretroviral drugs [1], partial escape from immune responses [2]–[4], the ability to switch tropism for target cells [5], and potential threats to new therapeutic strategies [6], [7]. The forces driving and influencing HIV evolution include Darwinian selection, limited population size, linkage, recombination, epistasis, spatial aspects, and dynamic factors (particularly due to the immune response). These factors, and the parameters that define them, can be difficult to discern. One of the most elusive parameters critically important for the rate of evolution in every medically relevant scenario is the “effective population number” (*N_e_*_ff_) (Figure 1). By definition, the census population size of HIV is the total number of infectious proviruses integrated into the cellular DNA of an individual at a given time. However, the genetically relevant *N_e_*_ff_ may differ substantially from the census population size. In this volume of *PLOS Genetics*, Pennings and colleagues [8] use new insights into “hard” and “soft” selective sweeps to estimate the effective population size of HIV.

The search for *N*_eff_ (and other HIV evolutionary parameters) has gone on for almost two decades, following every turn and hitting each pothole on the eventful road of HIV modeling [9]. The rapidity of resistance to monotherapy (in 1–2 weeks) was explained by the deterministic selection of alleles that preexist therapy in minute quantities [1]. The large numbers of virus-producing cells (∼10^8^) in the lymphoid tissue of experimentally infected macaques seemed to confirm this simple Darwinian selection model [10]. However, the Darwinian view has faced challenges. Tajima's “neutrality test” applied to HIV sequences in untreated patients assumed that selection was neutral and predicted much smaller “effective” populations, of *N*_eff_∼10^3^ [11]. Since Tajima's approach was designed to detect isolated selective sweeps at one or a few mutant sites—while HIV exhibits hundreds of diverse sites in vivo—two groups re-tested the result. A linkage disequilibrium (LD) test [12] and analysis of the variation in the time to drug resistance [13] arrived at the same value, *N*_eff_ = (5–10)×10^5^, for an average patient (with the mutation rate ∼10^−5^ per base). Such populations are sufficiently large for deterministic selection to dominate, yet not large enough to neglect stochastic effects altogether. The LD test [12] is affected by recombination, and HIV's recombination rate had not been well measured at that time. The recent measurement of 5×10^−6^ crossovers per base per HIV replication cycle in an average untreated individual [14]–[16] updates *N*_eff_ to (1–2)×10^5^, not far from the original value. A recent study of the pattern of diversity accumulation in early and late HIV infection confirms the range of *N*_eff_ [17]. However, all these estimates of *N*_eff_ are lower bounds.

Pennings et al. [8] continue this quest for an effective population size of HIV using a new method based on a theoretical calculation of the probability of multiple introductions of a beneficial allele at a site before it is fixed in a population [18]. The prediction does not depend on whether mutations are new or result from standing variation prior to therapy. The authors use sequence data obtained from 30 patients who failed suboptimal antiretroviral regimens, including efavirenz [19]—a non-nucleoside reverse transcriptase (RT) inhibitor (NNRTI)—and who exhibited a rise of drug-resistant alleles in RT. The sequence data reveal fixation of two alleles, both corresponding to an amino-acid replacement K103N. Pennings et al.'s analysis focuses on the genetic composition at RT codon 103 and the adjacent 500 nucleotides. Based on the changes in the genetic diversity in this region, 30 fixations are classified into “hard” selective sweeps with a single parental sequence, or “soft” sweeps with multiple parental sequences. Observing that both types of sweep occurred at similar frequencies (also confirmed by observations in other resistance codons), the authors predict *N*_eff_ = 1.5×10^5^, in agreement with the LD test.

Pennings et al. also discuss why “selectively neutral” methods based on synonymous diversity underestimate the population size. It is well known that a selection sweep lowers the diversity at linked sites (hence the term “sweep”) and any method assuming selective neutrality translates lower diversity to smaller *N*_eff_. The interesting part is the dynamic component of this effect. Pennings et al. demonstrate that rapid sweeps are followed by long periods when the diversity recovers at the linked sites (for synonymous sites, these periods are very long). From another angle, we can add that selection shortens the time to the common ancestor, which decreases the sequence divergence. The ancestral-tree argument is rather general and also applies to a large number of linked sites evolving under selection [20]–[23].

The previous estimates [12], [13], [17] were lower bounds on *N*_eff_. In contrast, the Pennings et al. study puts a number on *N*_eff_. However, this number (*N*_eff_ = 1.5×10^5^) raises a question: why is *N*_eff_ so far below the census population size of 10^8^ or more? Pennings et al. offer an elegant explanation of this relatively small *N*_eff_ in the spirit of the “traveling wave” approach [24]–[27]. They note that resistant alleles at different sites emerge against different fitness backgrounds. To be fixed, alleles conferring a small benefit must emerge in the most-fit genomes [28], [29]; hence, the effective *N*_eff_ for these alleles is small. Alleles with a larger beneficial effect can explore a larger fraction of population (larger *N*_eff_). Conceptually, this idea is quite correct; quantitatively, in the context of drug resistance, some problems arise. For example, the fitness benefit from a resistance mutation (under drug) is almost 100%, while the difference between the fittest and the average genome (in untreated patients) is a modest ∼10% [14]. Indeed, the average selection coefficient is quite small, ∼0.5% [14], [15].

There may be several other reasons for *N*_eff_<10^8^, as follows.

- By considering only 500 bases (∼5%) of the HIV genome, the study may underestimate the number of genetic backgrounds in which the resistant allele can be observed.
- *N*_eff_ is likely to vary in time—similar to viremia, which decays strongly after the onset of therapy and rebounds after its failure—and the placement of the inferred population size within the therapy time frame is unclear. Specifically, it is unclear from the empirical source [19] whether K103N mutations are generated before therapy (which is likely, considering that the mutation of interest decays very slowly in vivo in untreated patients and therefore has a low mutation cost [30]) or after therapy fails for another reason (see Figure 1 in [19]). In the first scenario, inferred *N*_eff_ = 10^5^ is the pretreatment number. In the second scenario, the pretreatment number must be much higher than 10^5^, since the replicating census population is reduced by a large factor (∼100) following initiation of therapy.
- Other factors, such as variation of the population number among patients and the spatial organization of the infected tissue [31] (both neglected in the test), may be relevant. Furthermore, the authors' calculations rely on the assumption of equal mutation rates for the two resistance mutations analyzed (both transversions). If the underlying rate of AAA to AAC is much greater than that of to AAT, the cited analysis would have underestimated the frequency of soft sweeps, yielding an underestimate of *N*_eff_.
- A significant complicating factor is the presence, in the parent study [19], of other drugs, particularly the nucleoside RT inhibitors (NRTIs) AZT and 3TC. In some cases, mutations conferring resistance to these drugs may have also contributed to failure (e.g., during the precursor monotherapy; see Figure 1 in [19]), and the requirement for these additional changes would have made the frequency of resistant strains much less than the estimate. For virus that escaped the combination treatment in the absence of NRTI mutations, replication was most likely occurring only in a fraction, or “sanctuary,” of cells that did not receive an inhibitory dose of these drugs. Either or both of these effects would have led to a potentially large underestimate of *N*_eff_. Indeed, a recent study of rapid NNRTI resistance, in SIV-infected monkeys treated with efavirenz monotherapy, used an ultrasensitive PCR assay to estimate the pre-therapy level of either K103N mutation as less than 0.0001% [32], implying a total replicating population of >10^6^.

For these reasons, the value *N*_eff_ = 1.5×10^5^ obtained in the study of Pennings et al. should probably still be regarded as a lower bound. At the same time, the study solidifies our understanding of HIV evolution as a Darwinian process and leads to important questions regarding the structure of HIV population, which are still waiting for new insights.
